# Supplementary material for: Predictors of Mortality in Patients with Infections Due to Carbapenem-Resistant Gram-Negative Bacteria
Source: Antibiotics (Basel). 2023 Jun 29;12(7):1130. doi: 10.3390/antibiotics12071130 (PMC10375996; doi:10.3390/antibiotics12071130)
Supplement: Supplementary file 1 [file antibiotics-12-01130-s001.zip › antibiotics-2438182-supplementary.pdf]

Supplementary material

Predictors of Mortality in Patients with Infections due to Carbapenem-Resistant Gram-Negative Bacteria

Table S1. Risk factors for the acquisition of CR-GNB and mortality

Variable	Total n=225 (100%)	Death at 90 days (33.8%)	Survivors during first 90 days n=149 (66.2%)	*p*
Chemotherapy in last 180 days, n (%)	48 (21.4)	26 (34.2)	22 (14.9)	0.001
Antibiotic use in last 180 days, n (%)	204 (9.7)	60 (90.8)	135 (90.6)	0.964
Health care utilization in last 180 days, n (%)	140 (62.2)	44 (57.9)	96 (64.4)	0.339
Resistant-bacterial infection in last 180 days, n (%)	62 (27.6)	16 (21.1)	46 (30.9)	0.119
Surgery in last 180 days, n (%)	74 (33.0)	19 (25.0)	55 (37.2)	0.067
ICU in last 180 days, n (%)	94 (42.0)	29 (38.2)	65 (43.9)	0.408
Intravascular devices use in last 180 days, n (%)	173 (76.9)	59 (77.6)	114 (76.5)	0.850
Antibiotic use at diagnosis, n (%)	195 (86.7)	69 (90.8)	126 (84.6)	0.194

ICU: intensive care unit.

Table S2. Laboratory values at the time of diagnosis of CR-GNB infection

**Laboratorios**	**Total n=225 (100%)**	**Death at 90 days (33.8%)**	**Survivors at 90 days n=149 (66.2%)**	***p***
Hemoglobin - g/dL, median (IQR)	9.1 (7.8-10.5)	8.4 (7.2-9.8)	9.5 (8.1-11.0)	0.0001
Leukocytes - x10³/µL, median (IQR)	9.1 (5.5 – 12.7)	10.8 (3.8-15.9)	8.5 (5.8-11.0)	0.0588
Total neutrophils - x10³/µL, median (IQR)	6.4 (2.3-9.9)	8.1 (1.7-11.8)	6.0 (2.8-8.7)	0.0963
Total lymphocytes - x10³/µL, median (IQR)	0.6 (0.2-1.1)	0.4 (0.1-0.9)	0.7 (0.3-1.2)	0.0077
Platelets - x10³/µL, median (IQR)	221 (107-357)	149 (27-276)	278 (182-378)	<0.0001
Glucose - mg/dL, median (IQR)	116 (97-147)	136 (115-158)	108 (94-136)	0.0001
Creatinine - mg/dL, median (IQR) n=204 (RRT excluded)	0.56 (0.41-0.93)	0.63 (0.42-1.15) n=65	0.54 (0.40-0.81) n=139	0.1289
Albumin - g/L, median (IQR) n=218	2.54 (2.19-2.98)	2.36 (1.93-2.79) n=76	2.68 (2.32-3.04) n=142	0.0003
Total bilirrubin - mg/dL, median (IQR) n=218	0.8 (0.5-1.8)	0.8 (0.5-2.5) n=76	0.7 (0.4-1.7) n=142	0.5178
C reactive protein mg/dL, median (IQR) n=209	13.6 (6.7-19.6)	16.0 (10.5-22.7) n=69	12.4 (5.2-17.8) n=140	0.0016

dL: deciliters, g: grams, **L**: liters, **mg**: milligrams, **IQR**: interquartile range, **RRT**: renal replacement therapy, **µL**: microliters

Table S3. Microbiological isolates and mortality

**Microbiological isolate**	**Total n=225 (100%)**	**Death at 90 days (33.8%)**	**Survivors at 90 days (66.2%)**	***p***
Enterobacterales	**122 (54.2)**	**41 (54.0)**	**81 (54.4)**	**0.953**

- *Escherichia coli*	*68/122*	*22/41*	*46/81*	
- *Klebsiella pneumoniae*	*21/122*	*7/41*	*14/81*	
- *Complejo Enterobacter*	*15/122*	*5/41*	*10/81*	
- *Raoultella sp*	*8/122*	*3/41*	*5/81*	
- *Klebsiella aerogenes*	*5/122*	*0/41*	*5/81*	
- *Citrobacter freundii*	*3/122*	*2/41*	*1/81*	
- *Klebsiella oxytoca*	*1/122*	*1/41*	*0/81*	
- *Serratia marcescens*	*1/122*	*1/41*	*0/81*	
Non-fermenting Gram-negative bacilli	**103 (45.8)**	**35 (46.1)**	**68 (45.6)**	
- *Pseudomonas aeruginosa*	*95/103*	*30/35*	*65/68*	
- *Acinetobacter baumanii*	*6/103*	*5/35*	*1/68*	
- *Pseudomonas sp*	*2/103*	*0/35*	*2/68*	

sp: species

Table S4. Microbiological isolates and types of infection

**Microbiological isolate**	**Bloodstream infection n=19 (%)**	**Respiratory tract infection n=88 (%)**	**Intraabdomi-nal infection n=85 (%)**	**Urinary tract infection n=28 (%)**	**Bone and soft tissue infection n=18 (%)**	**Other infection n=4 (%)**
Enterobacterales	14 (73.7)	36 (40.9)	50 (58.2)	16 (54.1)	12 (66.7)	2 (50)
- *E. coli*	*10/19*	*14/88*	*37/85*	*8/28*	*6/18*	*1/4*
- *K. pneumoniae*	*1/19*	*4/88*	*6/85*	*3/28*	*6/18*	*1/4*
- *C. Enterobacter*	*3/19*	*8/88*	*3/85*	*1/28*	*0/18*	*0*
- *Raoultella sp*	*0/19*	*3/88*	*2/85*	*3/28*	*0/18*	*0*
- *K. aerogenes*	*0/19*	*4/88*	*0/85*	*1/28*	*0/18*	*0*
- *C. freundii*	*0/19*	*2/88*	*1/85*	*0/28*	*0/18*	*0*
- *K. oxytoca*	*0/19*	*1/88*	*0/85*	*0/28*	*0/18*	*0*
- *S. marcescens*	*0/16*	*0/88*	*1/85*	*0/28*	*0/18*	*0*
Non-fermenting Gram-negative bacilli	5 (26.3)	52 (59.1)	35 (41.2)	12 (42.9)	6 (33.3)	2 (50)
- *Pseudomonas aeruginosa*	5/19	46/88	34/85	11/28	6/18	2/4
- *Acinetobacter baumanii*	0/19	5/88	0/85	1/28	0/18	0/4
- *Pseudomonas sp*	0/19	1/88	1/85	0/28	0/18	0/4

Table S5. Microbiological isolates and non-susceptibility frequency.

Microbiological isolate	TZP	C3G*	AK	CIP	TIGE	COL	ETP	IMP	MEM
**Enterobacterales**									
E. coli	56/59	60/66	3/66	61/66	1/59	0/58	66/67	46/67	51/68
K. pneumoniae	19/19	20/21	3/21	19/21	0/14	0/19	18/21	17/21	12/21
C. *Enterobacter*	6/13	8/15	0/15	3/15	0/12	0/10	7/15	6/15	0/15
Raoultella sp	6/6	1/8	0/8	7/8	0/6	0/6	5/7	6/7	2/8
K. aerogenes	2/5	2/5	0/5	1/5	1/5	0/2	2/5	3/5	0/5
C. freundii	3/3	2/3	0/3	2/3	0/3	0/2	3/3	2/3	0/3
K. oxytoca	1/1	0/1	0/1	1/1	0/1	0/1	1/1	0/1	1/1
S. marcescens	0/1	0/1	0/1	0/1	0/1		1/1		0/1
**Non-fermenting Gram-negative bacilli**									
Pseudomonas aeruginosa	39/86	35/93	20/93	32/91		4/81		88/91	88/94
Acinetobacter baumanii	5/6	5/6	5/6	5/6	2/6	0/3		4/6	4/6
Pseudomonas sp	1/2	1/2	0/1	1/1		0/2		2/2	2/2

AK: amikacin, **C3G**: third generation cephalosporins, **CIP**: ciprofloxacin, **COL**: colistin, **ETP**: ertapenem, **IMP**: imipenem, **MEM**: meropenem. **Sp**: species, **TIGE**: tigecycline, **TZP**: piperacillin/tazobactam,

*Ceftriaxone in the case of Enterobacterales and Ceftazidime in the case of non-fermenting Gram-negative bacilli.

Table S6. Microbiological isolates and presence of carbapenemases.

Microbiological isolate	mCIM n=126	eCIM n=79	OXA-48 N=88	KPC N=88	NDM N=88	GES N=88
**Enterobacterales**						
E. coli	48/63	33/48	11/52	1/52	20/52	3/52
K. pneumoniae	18/20	4/18	6/11	2/11	3/11	4/11
C. *Enterobacter*	4/13	0/4	1/6	1/6	3/6	0/6
Raoultella sp	7/7	0/7	4/4	0/4	0/4	1/4
K. aerogenes	1 / 2	0/1	0/1	0/1	0/1	0/1
C. freundii	1/1	0/1	0/0	0/0	0/0	0/0
K. oxytoca	0/0		1/1	0/1	0/1	0/1
S. marcescens	0/1		0/0	0/0	0/0	0/0
**Non-fermenting Gram-negative bacilli**						
Pseudomonas aeruginosa			0/13	0/13	0/13	4/13
**Total**	79	37	23	4	26	12

eCIM: EDTA-modified carbapenem inactivation method, **GES**: "Guiana extended spectrum" type enzyme, **KPC**: *Klebisella pneumoniae* carbapenemase, **mCIM**: carbapenem inactivation method, **NDM**: New Dheli Metallo-beta-lactamase, *OXA-48*: oxacillinase 48.

Table S7. Appropriate treatment and mortality at 90 days.

**Appropiate treatment**	**Total n=225 (100%)**	**Death at 90 days n=76 (33.8%)**	**Survivors at 90 days n=149 (66.2%)**	***p***
Yes, n (%)	196 (87.1)	57 (75.0)	139 (93.3)	<0.0001
No, n (%)	29 (12.9)	19 (25.0)	10 (6.7)	

Table S8. Type of appropriate antibiotic treatment received.

**Appropiate treatment**	**Total=196**
Carbapenem	44/196
Aminoglycoside	55/196
Piperacillin/tazobactam	31/196
Tigecycline	35/196
Trimethoprim/sulfamethoxazole	1/196
Ceftazidime	29/196
Ceftazidima/avibactam	8/196
Ceftolozane/tazobactam	1/196
Quinolones	5/196
Fosfomycin	9/196
Colistin	35/196

Table S9. Treatment duration by type of infection.

	**Total n=225 (100%)**	**Death at 90 days n=76 (33.8%)**	**Survivors at 90 days n=149 (66.2%)**	***p***
Treatment duration - days, median (IQR)	10 (7-15)	8 (1-12)	10 (7-15)	0.0031
Bloodstream infections	*10 (7-17)*	*15 (0-23)*	*10 (8-15)*	*0.6468*
Respiratory tract infections	*8 (5-13)*	*7 (0-10)*	*10 (7-14)*	*0.0009*
Intraabdominal infections	*10 (7-21)*	*8 (5-14)*	*11 (7-24)*	*0.1643*

Urinary tract infections	*10 (6-13)*	*10 (10-11)*	*9 (6-14)*	*0.5258*
Bone and soft tissue infections	*11 (7-38)*	*11 (6-38)*	*13 (8-41)*	*0.8239*
Others	*7 (4-11)*	*-*	*7 (4-11)*	*-*

IQR: interquartile range

Table S10. Bivariate analysis for primary outcome.

**Variable**	**HR (CI 95%), *p***

Male gender	1.37 (0.84-2.23), 0.206
Age	1.02 (1.00-1.03), 0.01
**CR-GNB infection**	
- Bloodstream infection	1.35 (0.65-2.81), 0.423
- Respiratory tract infection	1.93 (1.23-3.03), 0.004
- Intraabdominal infection	0.36 (0.21-0.63), <0.001
- Urinary tract infection	0.43 (0.17-1.07), 0.69
- Bone and soft tissue infection	2.17 (1.11-4.23), 0.022
**Comorbidity**	
- Charlson index >3	2.08 (2.33-3.27), 0.001
- COVID-19	0.86 (0.50-1.48), 0.595
- Obesity	0.86 (0.49-1.50), 0.592
- Diabetes	1.16 (0.68-1.94), 0.563
- Heart disease	1.88 (1.14-3.08), 0.013
- Hypertension	1.21 (0.76-1.93), 0.413
- COPD	1.91 (0.60-6.07), 0.271
- Immunosuppression	1.86 (1.18-2.92), 0.007
- Iatrogenic bile duct injury	0.51 (0.24-1.12), 0.095
- Liver cirrhosis	2.58 (0.81-8.20), 0.108
- Renal replacement therapy	1.95 (1.03-3.70), 0.040
- Cerebrovascular disease	1.83 (0.67-5.01), 0.241
- Urological disorders	0.42 (0.15-1.15), 0.092
- Tracheostomy carriers	0.80 (0.40-1.60), 0.535
- Mechanical ventilation at diagnosis	1.78 (1.12-2.83), 0.015
- Septic shock at the time of diagnosis	2.75 (1.75-4.33), <0.0001
- Bacterial coinfection	0.99 (0.63-1.56), 0.979
- Secondary bacteremia	0.84 (0.49-1.42), 0.150
**Risk factor for CR-GNB infections**	
- Chemotherapy in last 180 days	2.12 (1.32-3.40), 0.002
- Antibiotic use in last 180 days	0.96 (0.44-2.09), 0.918
- Health care utilization in last 180 days	0.78 (0.49-1.22), 0.285
- Resistant-bacterial infection in last 180 days	0.62 (0.36-1.08), 0.092
- Surgery in last 180 days	0.60 (0.36-1.01), 0.052
- ICU in last 180 days	0.84 (0.53-1.33), 0.455
- Intravascular devices use in last 180 days	0.99 (0.58-1.70), 0.972
- Antibiotic use at diagnosis	1.66 (0.76-3.62), 0.200
**Patient location at diagnosis**	
ICU	2.08 (1.32-3.26), 0.001
**Laboratorios, mediana**	
- Hemoglobin <10 g/dl	2.56 (1.46-4.51), 0.001

- Leukocytes <3,900 x10³/μL	1.76 (1.06-2.93), 0.030
- Total neutrophils <1,500 x10³/μL	0.9 (0.50-1.7), 0.779
- Total lymphocytes <800 x10³/μL	1.52 (0.94-2.45), 0.087
- Platelets <150,000 x10³/μL	2.95 (1.88-4.64), <0.0001
- Glucose >200 mg/dl	2.31 (1.06-5.03), 0.036
- Creatinine >1.2 mg/dl<br>n=204 (excluído TSR)	1.81 (1.03-4.19), 0.039
- Albumin <3.5 mg/dl<br>n=218	2.52 (1.58-4.02), <0.001
- Total bilirrubin >1.2 mg/dl<br>n=218	1.17 (0.74-1.85) 0.504
- C reactive protein >10 mg/dl<br>n=209	2.20 (1.27-3.80), 0.005
Non-fermenting Gram-negative bacilli	1.05 (0.67-1.65), 0.832
Appropiate treatment	0.26 (0.15-0.44), <0.0001
Acute kidney injury within the first 10 days of treatment	2.37 (1.43-3.91), 0.001
Mechanical ventilation at diagnosis	2.90 (1.60-5.28), <0.0001
ICU stay after diagnosis	2.57 (1.48-4.47), 0.001
Carbapenemase-carrier Enterobacterales (mCIM positive)<br>n=107	1.12 (0.54-2.32), 0.756
Metallo-ß-lactamase vs serine-carbapenemase in Enterobacterales<br>n=72	1.20 (0.53-2.73), 0.440

CI: confidence interval, **CR-GNB**: carbapenem-resistant Gram-negative bacilli, **COPD**: chronic obstructive pulmonary disease**, CVD**: cerebrovascular disease, **HR**: hazard ratio, **mCIM**: carbapenem inactivation method, **ICU**: intensive care unit.

Considerations for the multivariate analysis model construction

Charlson was not included in the model since this variable includes age and comorbidities. COVID-19 was not included in the model due to interactions with obesity, diabetes, and upper respiratory tract infection. COPD was not included due to its interaction with heart disease. Iatrogenic bile duct injury interacted with abdominal sepsis was not included. Stroke interacted with diabetes mellitus was not included. Urological alterations or tracheostomy carriers interacted with urinary and respiratory infection, respectively, and were not included. Bacterial co-infection was not included at the investigators' discretion as failure of appropriate treatment is not expected. Secondary bacteremia was excluded because it was exclusive to primary bloodstream infections. Risk factors for acquiring BGNRC infections (e.g., prior chemotherapy, etc.) interacted with comorbidities (eg immunosuppression) and were not included. Laboratory parameters were not included as it is impossible to account for the variability of results over time and interactions with comorbidities. According to the prespecified analysis plan, secondary outcomes were not included

in the predictive model of the primary outcome. Carbapenemase phenotype and genotype were not included, as testing was not performed on the entire cohort
